# Feature-based multiple models improve classification of mutation-induced stability changes

**DOI:** 10.1186/1471-2164-15-S4-S6

**Published:** 2014-05-20

**Authors:** Lukas Folkman, Bela Stantic, Abdul Sattar

**Affiliations:** 1 Institute for Integrated and Intelligent Systems, Griffith University, Brisbane, Australia; 2 NICTA - National ICT Australia

## Abstract

**Background:**

Reliable prediction of stability changes in protein variants is an important aspect of computational protein design. A number of machine learning methods that allow a classification of stability changes knowing only the sequence of the protein emerged. However, their performance on amino acid substitutions of previously unseen non-homologous proteins is rather limited. Moreover, the performance varies for different types of mutations based on the secondary structure or accessible surface area of the mutation site.

**Results:**

We proposed *feature-based multiple models *with each model designed for a specific type of mutations. The new method is composed of five models trained for mutations in exposed, buried, helical, sheet, and coil residues. The classification of a mutation as stabilising or destabilising is made as a consensus of two models, one selected based on the predicted accessible surface area and the other based on the predicted secondary structure of the mutation site. We refer to our new method as *Evolutionary, Amino acid, and Structural Encodings with Multiple Models *(EASE-MM). Cross-validation results show that EASE-MM provides a notable improvement to our previous work reaching a Matthews correlation coefficient of 0.44. EASE-MM was able to correctly classify 73% and 75% of stabilising and destabilising protein variants, respectively. Using an independent test set of 238 mutations, we confirmed our results in a comparison with related work.

**Conclusions:**

EASE-MM not only outperformed other related methods but achieved more balanced results for different types of mutations based on the accessible surface area, secondary structure, or magnitude of stability changes. This can be attributed to using multiple models with the most relevant features selected for the given type of mutations. Therefore, our results support the presumption that different interactions govern stability changes in the exposed and buried residues or in residues with a different secondary structure.

## Background

A non-synonymous single nucleotide polymorphism (SNP) in a coding region of DNA results in a single amino acid polymorphism (a mutation) in a protein sequence. The ability to predict how such an amino acid substitution affects the stability of a protein is an important aspect of computational protein design. Moreover, it has been shown that disease-associated protein variants are often characterised by mutation-induced stability changes [1]. Therefore, an improved prediction of stability changes may help us deepen our understanding of the relationship between protein mutations and inherited diseases.

With the immense amounts of data about protein variants coming from the genome sequencing projects, computational methods, being fast and inexpensive, became convenient tools to study stability changes. These computational approaches can be categorised as *energy-based *and *training-based *methods. While *energy-based *methods use physical, statistical, or empirical energy functions to estimate the stability change from the protein's three-dimensional structure [2-9], *training-based *methods are trained on the experimental data from the ProTherm database [10] employing machine learning algorithms [11-26]. Interestingly, a number of the training-based methods allow for a prediction knowing only the sequence of a protein [17-26]. Since there is a large gap between the number of known protein sequences and experimentally determined three-dimensional structures, we devote our interest to these *sequence-based *methods in this work.

While a number of the sequence-based methods were able to report a high prediction accuracy, the results from an assessment study showed that the performance of three evaluated methods was much lower on an independent test set [27]. There, only the mutations from new additions to the ProTherm database were used for testing. This finding correlates with the results reported in our recent work [26]. We found that the prediction performance of three methods in our comparison was relatively low when evaluated solely on mutations in proteins with low sequence similarity to the training set. To improve prediction performance on non-homologous proteins, we proposed a method based on evolutionary and structural encodings with amino acid parameters. While the new method was able to outperform related work, the analysis revealed that the performance for exposed residues was considerably lower than for buried ones. Similarly, mutations in coil residues appeared to be more difficult to predict than the ones in *α*-helices and *β*-sheets.

In this work, we followed the observation that prediction performance differs among various types of mutations based on the accessible surface area or secondary structure. By employing feature selection, we built specialised *feature-based multiple models*, each dedicated to a specific type of mutations. Our results show that this methodology improves two-class prediction of stability changes. Moreover, a consensus approach combining two methods with multiple models (one based on the accessible surface area and the other on the secondary structure) yielded further improvements. Analysis of our results revealed that the new method delivers more balanced predictions than our previous work for mutations in residues with a different secondary structure and solvent accessibility as well as for different magnitudes of stability changes. Finally, our new method achieved a favourable performance in a comparison with related work using an independent test set of 238 mutations. We refer to the new method as *Evolutionary, Amino acid, and Structural Encodings with Multiple Models *(EASE-MM).

## Methods

### Feature-based multiple models

We built and compared four different machine learning methods for the two-class (stabilising and destabilising) prediction of stability changes. The first one (referred to as EASE-AA_2_) was an extension of our previous work (EASE-AA: *Evolutionary And Structural Encodings with Amino Acid parameters *[26]). EASE-AA_2 _employed a single support vector machine (SVM) model with predictive features selected using a greedy feature selection algorithm. We used a range of features describing evolutionary conservation, amino acid parameters, and structural properties. Next, we developed two methods, each composed of several SVM models. The motivation here was to make each model specialised for different types of mutations. The first method (EASE-ASA) was composed of two models based on different accessible surface area (ASA) categories (exposed and buried). The second method (EASE-SS) was composed of three models based on different secondary structure (SS) types (*α*-helix, *β*-sheet, and coil). Finally, we built a consensus method combining the predicted probabilities of the two methods with multiple models. We refer to the consensus method as EASE-MM.

**Figure 1:**
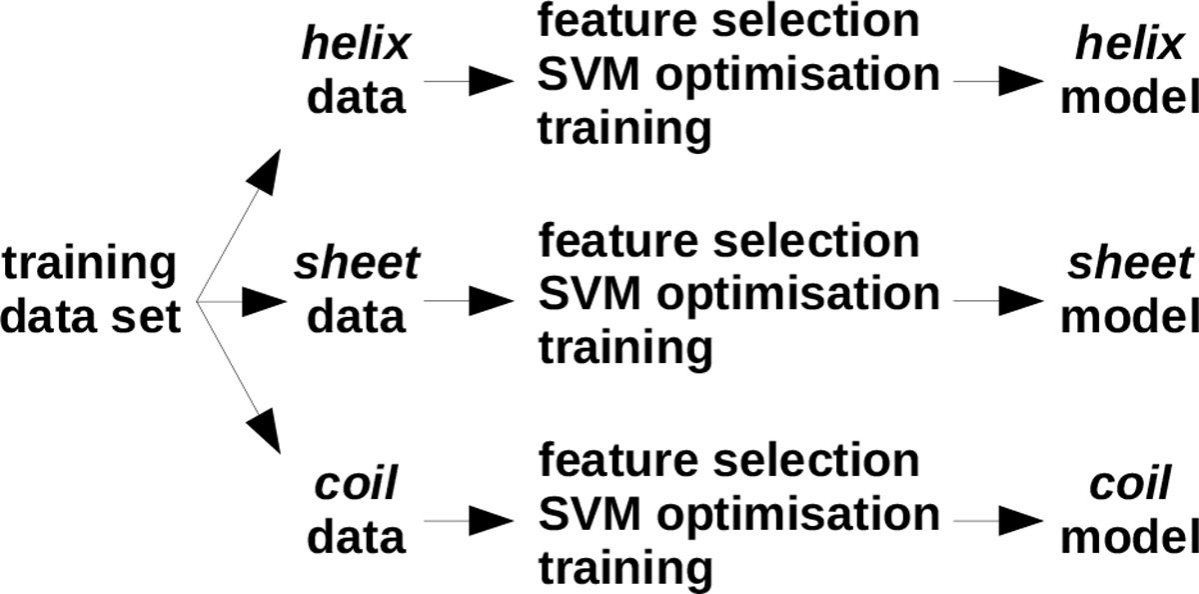
**Design of feature-based multiple models**. The design of EASE-SS is shown, however, the same applies to EASE-ASA. First, the data was divided according to the predicted secondary structure (accessible surface area). Then, relevant predictive features were selected using a greedy feature selection algorithm. SVM parameters were optimised using a grid search. Finally, the predictive models were trained.

Figure 1 illustrates the design of EASE-SS, however, the same applies to EASE-ASA. First, we partitioned the training data according to the secondary structure (accessible surface area) of the mutation site. Second, we used feature selection to select a relevant combination of features for the given data partition. Next, the SVM parameters were optimised and the model was trained for classification of stability changes. Since we aimed to design strictly *sequence-based *methods, the secondary structure and two categories of accessible surface area were predicted with SPINE-X [28] and ACCpro [29], respectively. A solvent accessibility threshold of 25% was used for classifying residues as exposed or buried. This threshold resulted in a well-balanced partitions of 785 exposed and 891 buried mutations. Further discussion on the solvent accessibility thresholds can be found in the next section (Results and discussion).

**Figure 2:**
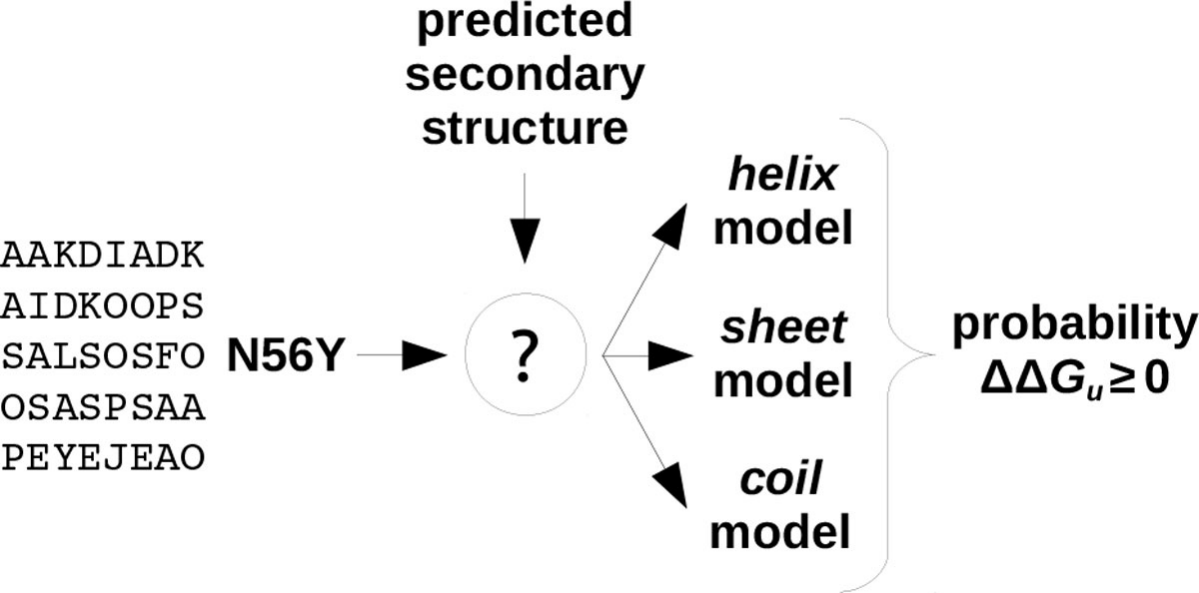
**Classification with feature-based multiple models**. The prediction process is shown for EASE-SS, however, the same applies to EASE-ASA. First, the secondary structure (accessible surface area) is predicted. Next, the relevant model is selected and used for prediction. That is, if the mutation is in a helical (exposed) residue, only the helix (exposed) model is used.

Figure 2 illustrates how a prediction is performed using EASE-SS (the same applies to EASE-ASA). Given the inputs (protein sequence and amino acid substitution), the secondary structure (accessible surface area) is predicted first. Then, the relevant model is selected. That is, if the mutation is in a helical (exposed) residue, only the *helix *(*exposed*) model is used. The output is a predicted probability of the mutation to be stabilising. Finally, for the case of the consensus method (not shown in the figure), prediction probability *P *is the average of the probabilities predicted with EASE-ASA and EASE-SS:

(1) $$P_{EASE-M\;M}=\frac{1}{2}\left(P_{EASE-ASA}+P_{EASE-SS}\right)$$

### Predictive features

For machine learning classification of stability changes, each mutation needs to be encoded with a number of predictive features. We considered a range of features describing the evolutionary conservation, amino acids parameters, and structural properties.

#### Evolutionary features

We used two evolutionary features: SIFT *score *(also employed in our previous work [25,26]) and the *difference of mutation and conservation likelihood *(Δ*M*). SIFT [30] predicts whether a mutation affects the function of a protein. It is calculated from a scaled probability matrix of possible amino acid substitutions generated from a multiple sequence alignment of related sequences. SIFT scores range from 0 to 1 where scores below 0.05 are predicted as deleterious mutations. We ran SIFT using the Swiss-Prot and TrEMBL databases with sequences more than 90% identical to the query removed.

Feature Δ*M *expresses the difference of likelihoods of the introduced and deleted amino acids to appear in the alignment of homologous sequences of the target protein. To calculate this feature, three iterations of PSI-BLAST [31] in default configuration were used to search the NCBI non-redundant database. Then, the likelihood scores were extracted from the last position specific scoring matrix (PSSM). The scores were divided by 10 for normalisation so that most values fell within the range of *−*1 and 1.

#### Amino acid parameters

A variety of different amino acid parameters were introduced for the prediction of stability changes [12,14,16,20,23]. In our previous work [26], we adopted seven representative parameters including *hydrophobicity, volume, polarisability, isoelectric point, helix tendency, sheet tendency*, and a *steric parameter *(graph shape index). These parameters were first introduced in [32] and later applied to prediction of secondary structure [28]. In this work, we included another 4 parameters: *flexibility *[33], *compressibility, bulkiness*, and *equilibrium constant with reference to the ionisation property of COOH group *[34]. We included these parameters because they were found as one of the best determinants to stability changes in the study of 48 physical-chemical, energetic, and conformational amino acid properties [12,14]. We normalised all 11 parameters to fall within the range of *−*0.9 and 0.9. The normalised values of the 11 parameters are available in Additional file 1.

We encoded each of the amino acid parameters as two distinct predictive features. The first one was equal to the difference between the amino acid parameters for the introduced and deleted amino acids (denoted as Δ). The other predictive feature described the mutation site environment as the mean, minimum, and maximum of the parameter values for a window of six neighbouring residues. We considered neighbourhood windows of up to a length of 18 and found that six neighbours performed optimally.

#### Structural features

Since structural information is not available in the case of *sequence-based *prediction of stability changes, we employed *predicted *structural features. We used the multistep neural network method SPINE-X [28] for the prediction of *secondary structure probabilities*. Also, the real value of the *relative accessible surface area *of each mutation site was predicted with SPINE-X. For the prediction of the *disorder probability*, we used the neural network method SPINE-D [35]. These three predicted structural features were also used in our previous work [25,26].

### Feature selection

We considered a range of predictive features and applied feature selection to design specialised models for 1) exposed and buried residues (EASE-ASA), and 2) helical, sheet, and coil residues (EASE-SS). Also, the single-model method (EASE-AA_2_) was designed employing the same feature selection procedure. We used *sequential forward floating selection *(SFFS) [36] which is a variation of a commonly adopted *sequential forward selection *(SFS) [37]. SFS works by iteratively adding the best-performing feature to a set of features *S*. Initially, *S *is empty. Every iteration, the best-performing feature *f *is selected as the one for which *S *∪ {*f*} achieves the highest prediction performance. The SFFS algorithm starts with SFS but at the end of every iteration, features are iteratively removed if this can further improve prediction performance. Thus, the number of features in *S *is not monotonously increasing because the search is 'floating' up and down. We ran SFFS until 10 features were added to *S *and stored all visited combinations of features. Finally, we selected the ultimately best-performing of the visited combinations. As a measure of prediction performance, the area under the receiver operating characteristic curve (commonly denoted as AUC) was used.

To verify the significance of the features selected with SFFS, we performed feature selection also with the *stability selection *algorithm [38]. In stability selection, the data sample of size *n *is randomly sub-sampled to size *n/*2. Then, an 'inner' feature selection algorithm is applied. The whole procedure is repeated *k*-times, each time with a different data sample. Features which are repeatedly selected contribute towards the final set of predictive features. We used SFS as the 'inner' algorithm and set *k *= 100.

### Support vector machines

Support vector machines (SVM) [39] are machine learning algorithms which can approximate non-linear functions by mapping the inputs to a high-dimensional feature space using a kernel function and then, solving a linear problem by finding a maximum margin separating hyperplane. We used the radial basis kernel function and implemented our method with the LIBSVM library [40].

To optimise the SVM performance, the regularisation parameter *C* and the radial basis kernel width parameter *γ *need to be set. If the number of positive and negative examples in the data set is unbalanced, SVM can be further optimised by setting the weight (*w*) of the penalty for a training error on positive examples. We optimised these SVM parameters by running a *grid search *using 10-fold cross-validation. In the grid search, we considered all possible combinations of *C *∈ {2^*−*5^, 2^*−*3^, . . . , 2^7^}, *γ *∈ {2^−7^, 2^−5^, . . . , 2^1^}, and *w *∈ {1, 1.5, 2, 2.5, 3}.

### Data sets

We compiled a data set of free energy stability changes from the ProTherm database [10] (February 2013). There, a stability change is defined as the difference in the unfolding free energy: ΔΔ*G_u_*[kcal mol^−1^] = Δ*G_u_*(*mutant*) *− *Δ*G_u_*(*wild*-*type*). Hence, we designated the positive and negative examples of the classification problem as the stabilising (ΔΔ*G_u _≥ * 0) and destabilising (ΔΔ*G_u _<* 0) mutations, respectively.

We extracted 3,329 mutations with listed stability changes and cross-checked all the sources where the measurements came from. We found that incorrect values (mostly the sign of ΔΔ*G_u_*) had been entered from at least 18 sources. We corrected stability changes for all relevant (*>*230) mutations in the extracted data set. Next, we removed all duplicate entries of the same amino acid substitutions (different concentrations of chemicals, stability changes of the protein intermediate state, etc.). If several measurements of the same mutation under the *same *experimental conditions were present, we averaged the stability changes and kept only a single entry. If several measurements of the same mutation under *different *experimental conditions were present, we kept only the measurement closest to the physiological pH 7.

Finally, we identified 74 clusters of proteins with more than 25% sequence similarity using BLASTCLUST [41]. If there were several measurements of the same amino acid substitution within a single cluster, we kept only the measurement closest to the physiological pH 7. This process yielded a non-redundant data set containing 1,914 mutations of 95 different proteins grouped into 74 non-homologous clusters.

To perform an independent comparison with related work, we separated all proteins with less than 25% sequence similarity to the data set used for the training of I-Mutant2.0 [17]. This procedure yielded 25 proteins with 238 mutations which we kept as an independent test set (S238). The remaining data of 1,676 mutations in 70 different proteins (S1676) were used for the development of our methods. Both S238 and S1676 data sets are available in Additional file 2.

### Evaluation

We used *unseen-protein *10-fold cross-validation on the S1676 data set to design our methods, select relevant features, and perform a comparison with our previous work (EASE-AA) [26]. In the *unseen-protein *cross-validation, we ensured that all mutations of any cluster of homologous proteins were contained within a single fold. Also, we kept the ratio of stabilising and destabilising examples reasonably similar among the folds. We repeated our experiments 100 times (each time with randomly generated folds) and averaged the results.

The *unseen-protein *cross-validation was previously used for the evaluation of a method for the prediction of deleterious mutations [42]. A similar procedure, *unseen-residue *cross-validation, was used for the evaluation of a *three-state *stability changes prediction method [21]. In our recent work [26], we compared both *unseen-residue *and *unseen-protein *cross-validation with the commonly-used random cross-validation. There, we concluded that the *unseen-protein *cross-validation provides the most robust estimate of the prediction performance. This is because predictive features cannot be selected as 'proxies' to specific residues or proteins.

For an independent comparison with related work, we used the S238 data set. This data was *not *used for the SVM parameters optimisation nor feature selection. Importantly, the sequence similarity between S1676 and S238 was less than 25%. To achieve a fair comparison with related work, we optimised prediction thresholds of all compared methods to yield a maximum Matthews correlation coefficient (MCC). MCC is a measure of prediction performance that provides more relevant information than classification accuracy (Q_2_) in cases when the data set is severely biased towards one class of examples. Since destabilising mutations prevail in the available experimental data, 72% and 81% of mutations were destabilising in the S1676 and S238 data sets, respectively.

Regarding evaluation measures, we assessed the overall prediction performance in terms of the receiver operating characteristic (ROC) curves and the area under the ROC curve (AUC). A ROC curve plots the true positive rate (sensitivity) as a function of the false positive rate (100% *− *specificity) at different prediction thresholds. Furthermore, we calculated Matthews correlation coefficient (MCC), classification accuracy (Q_2_), sensitivity (Se), specificity (Sp), positive predictive value (PPV), and negative predictive value (NPV):

(2) $$\text{MCC}=\frac{T\;P\times T\;N-F\;P\times F\;N}{\sqrt{\left(T\;P+F\;P\right)\;\left(T\;P+F\;N\right)\;\left(T\;N+F\;P\right)\;\left(T\;N+F\;N\right)}}$$

(3) $${\text{Q}}_{\text{2}}=\frac{T\;P+T\;N}{T\;P+F\;P+T\;N+F\;N}\;\times \;100$$

(4) $$\text{Se}=\frac{T\;P}{T\;P+F\;N}\;\times \;100$$

(5) $$\text{Sp}=\frac{T\;N}{T\;N+F\;P}\;\times \;100$$

(6) $$\text{PPV}=\frac{T\;P}{T\;P+F\;P}\;\times \;100$$

(7) $$\text{NPV}=\frac{T\;N}{T\;N+F\;N}\;\times \;100,$$

where *TP, TN, FP*, and *FN *refer to the number of true positives, true negatives, false positives, and false negatives, respectively.

## Results and discussion

Our main interest was to asses whether a method with *feature-based multiple models *can improve prediction performance compared to methods employing only a single model. To explore different ideas, we designed two methods with multiple models: EASE-ASA (composed of two models for exposed and buried residues) and EASE-SS (composed of three models for helical, sheet, and coil residues). We also built a consensus method (EASE-MM) of the former two. The most relevant features for each model were chosen using sequential forward floating selection (SFFS). We compared the 10-fold cross-validation performance (data set S1676) of the three methods with multiple models and two single-model methods (EASE-AA and EASE-AA_2_). While EASE-AA is our previous work [26], EASE-AA_2 _was designed using the same feature selection procedure as the methods with multiple models. Next, we analysed the significance of the selected features and investigated performance of our methods for different types of mutations. Finally, we performed a comparison with related work on an independent test set of 238 mutations.

### Cross-validation performance

We performed 100 replications of 10-fold cross-validation on the S1676 data set. Table 1 summarises the averaged results. Our previous work, EASE-AA, yielded the lowest prediction performance with a Matthews correlation coefficient (MCC) of 0.35. EASE-AA_2 _achieved only a marginal absolute improvement of 0.03 in MCC reaching a value of 0.38. We observed more notable improvements for the methods employing multiple models. EASE-ASA and EASE-SS reached MCC values of 0.40 and 0.42, respectively. These results constitute respective relative improvements of 14% and 20% (absolute improvements of 0.05 and 0.07) compared to our previous work (EASE-AA). The consensus method combining the predicted probabilities of EASE-ASA and EASE-SS yielded further improvements. EASE-MM achieved an MCC of 0.44 which represents a relative improvement of 26% (an absolute improvement of 0.09) compared to EASE-AA. Compared to EASE-AA_2_, the relative (absolute) improvement was 16% (0.06). While EASE-MM did not improve on EASE-AA_2_'s specificity (the accuracy on negative examples), negative predictive value was improved. This means that EASE-MM did not 'over-predict' destabilising mutations as much as EASE-AA_2_.

**Table 1:** Cross-validation performance (data set S1676) of our previous work, the single-model method, and the three methods with multiple models.

Method	AUC	MCC	Q_2_	Se	Sp	PPV	NPV
EASE-AA	0.76	0.35	67.11	74.93	64.22	43.68	87.37
EASE-AA_2_	0.77	0.38	72.67	65.54	75.31	49.58	85.51
EASE-ASA	0.80	0.40	72.62	71.07	73.19	49.55	87.23
EASE-SS	0.80	0.42	73.57	72.13	74.11	50.79	87.77
EASE-MM	0.82	0.44	74.71	73.14	75.28	52.30	88.33

EASE-MM is a consensus method of EASE-ASA and EASE-SS

**Figure 3:**
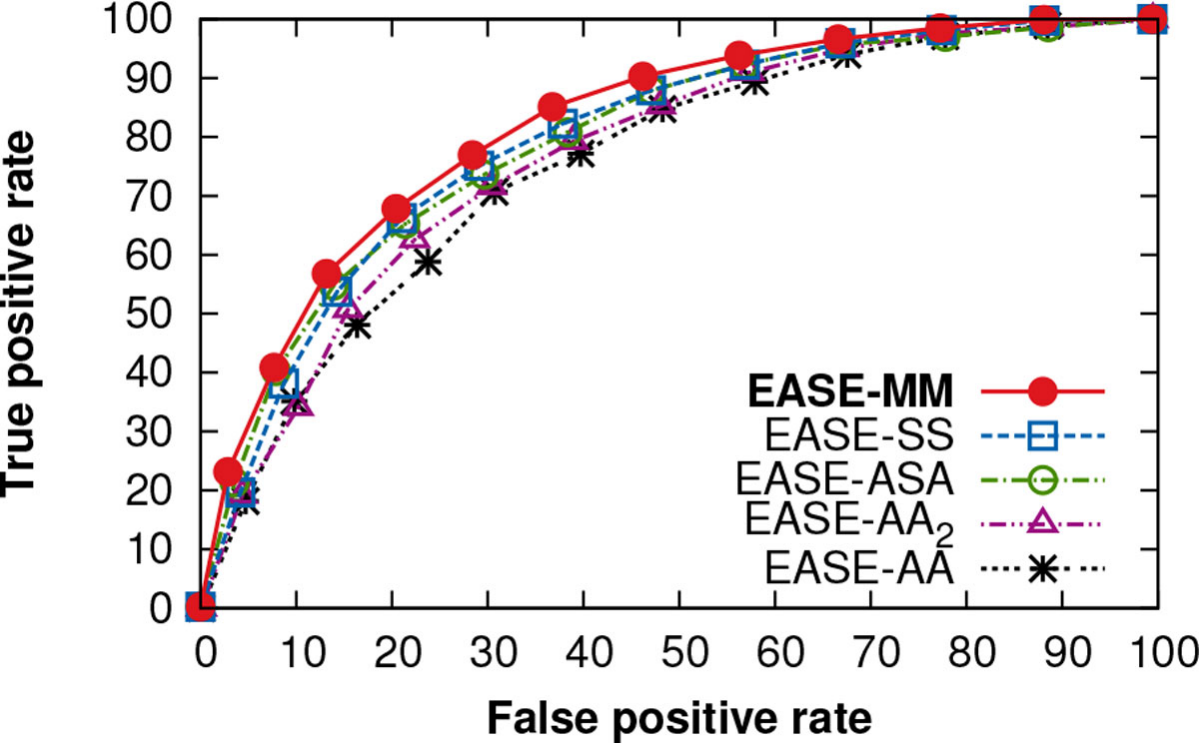
**ROC curves performance of the three methods with multiple models, the single-model method, and our previous work**. The true positive rate is shown as a function of the false positive rate at different prediction thresholds. These are cross-validation results with the S1676 data set. EASE-MM, EASE-SS, EASE-ASA, EASE-AA_2_, and EASE-AA achieved the area under the ROC curve (AUC) of 0.82, 0.80, 0.80, 0.77 and 0.76, respectively.

The receiver operating characteristic (ROC) curves in Figure 3 compare the true positive rate of the five methods as a function of the false positive rate at different prediction thresholds. The figure demonstrates the benefit in terms of the number of correctly predicted positive examples upon employing the methods with multiple models. EASE-ASA, EASE-SS, and EASE-MM achieved the area under the ROC curve (AUC) of 0.80, 0.80, and 0.82, while EASE-AA and EASE-AA_2 _yielded an AUC of 0.76 and 0.77, respectively. This represents a relative improvement of 6% (an absolute improvement of 0.05) for EASE-MM compared to EASE-AA_2_.

We estimated the statistical significance of the improvements yielded by the methods with multiple models over the 100 replications of cross-validation using a student *t*-test. The null hypothesis stated that there was no statistical difference in the MCC (AUC) for EASE-MM (EASE-ASA, EASE-SS) compared to EASE-AA_2 _(EASE-AA). The *p*-values associated with this null hypothesis were all less than 0.0001. Also, EASE-MM's improvements compared to EASE-ASA (EASE-SS) were statistically significant (*p*-values *<* 0.0001).

### Significance of the selected predictive features

**Figure 4:**
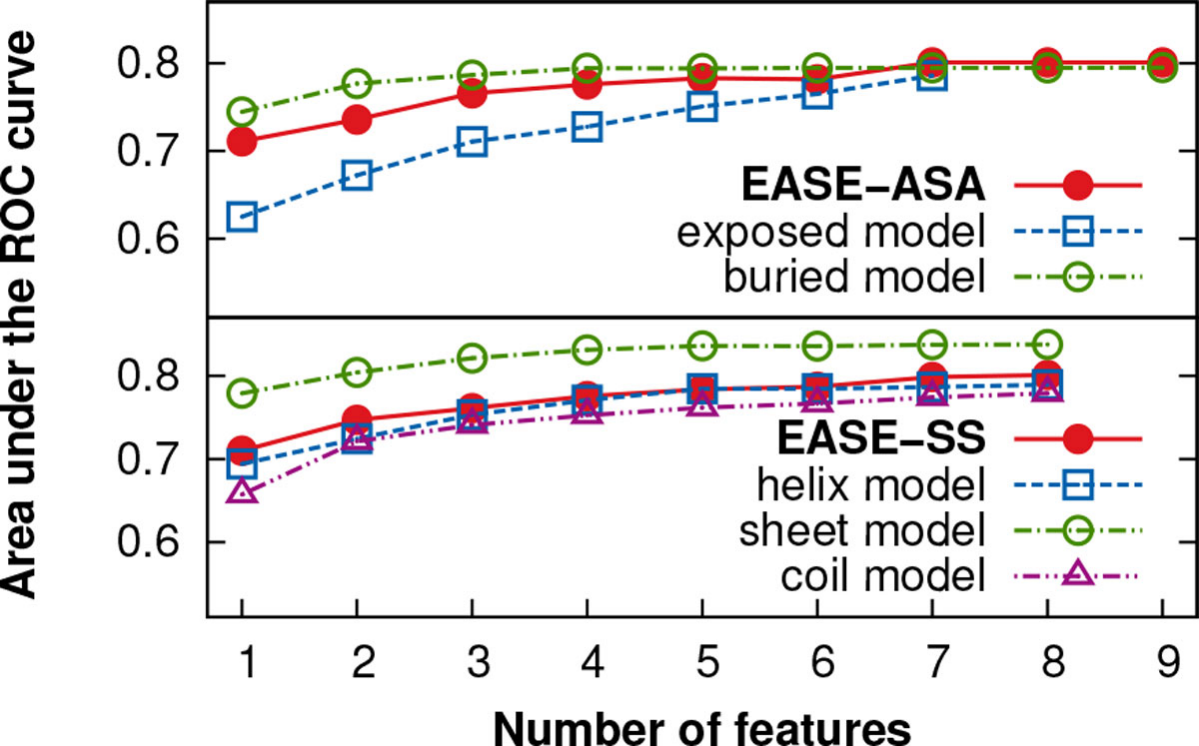
**Feature selection progress**. The area under the ROC curve (AUC) is shown as a function of the number features selected during feature selection. The prediction performance of the different models became more balanced as the feature selection progressed. These are cross-validation results with the S1676 data set.

We used the sequential forward floating selection (SFFS) to automatically select the combinations of the most relevant predictive features. While seven and nine features were selected for the *exposed *and *buried *models of EASE-ASA, respectively, each model of EASE-SS (*helix, sheet*, and *coil*) was composed of eight features. Figure 4 shows the area under the ROC curve (AUC) as a function of the number features selected with the SFFS algorithm. This figure illustrates the motivation for our work well. While at the early stages of feature selection, there is a notable gap between the prediction performance for mutations in the exposed and buried residues, as feature selection progresses, the performance becomes balanced for both categories. A similar trend, however not as pronounced, can be seen for the case of EASE-SS. Additional file 3 lists the final combinations of features implemented in EASE-ASA, EASE-SS, and EASE-AA_2_.

We analysed which features were most often selected across the five different models of EASE-ASA and EASE-SS. SFFS selected feature SIFT *score *for each of the five models. Feature Δ *hydrophobicity *was selected in all but the *sheet *model. The third most often occurring feature was the *relative accessible surface area*. It is of interest to inspect which features were the most specific for each of the models. For instance, amino acid attributes Δ *helix tendency *and Δ *sheet tendency *were chosen for the *helix *and *sheet *models, respectively. These features are indeed relevant specifically to the two models because they express the change in the preference of the given secondary structure type. Feature Δ *volume *was selected for both *helix *and *sheet *but not for the *coil *model. We conjecture that an increase in the side-chain volume may induce strain in the backbone of the protein in regions with a well-defined secondary structure but can be better tolerated in a coil region. Regarding the two models of EASE-ASA, features Δ *isoelectric point *and Δ *polarisability *were selected for the *buried *but not for the *exposed *model.

While SFFS was effective in finding the most relevant combination of predictive features, it does not provide a ranking of the individual features. This is because, as the feature selection progresses, even a significant feature can be removed if it does not perform well in combination with the others. We employed stability selection to analyse the significance of individual features. We implemented stability selection as 100 replications of the basic sequential forward selection (SFS) each time executed on a randomly sub-sampled S1676 data set (data was not partitioned for different types of mutations in this experiment). From the 100 results, we estimated the significance of each feature as the probability of being selected. Table 2 lists the nine most significant features. Evolutionary feature SIFT *score*, amino acid parameter Δ *hydrophobicity*, and structural property *relative accessible surface area *seem to be the most significant. This finding agrees with the most often occurring features across the five models of EASE-ASA and EASE-SS.

**Table 2:** The nine most significant features according to stability selection on the S1676 data set.

Feature	Significance
SIFT score	1.00
Δ hydrophobicity	0.97
relative accessible surface area	0.86
disorder probability	0.81
Δ compressibility	0.80
Δ polarisability	0.58
volume (mean, min, max)	0.54
Δ isoelectric point	0.53
secondary structure probabilities	0.47

Δ refers to the change between the introduced and deleted amino acids; (mean, min, max) was calculated for a window of six neighbouring residues; significance denotes the probability of being selected with stability selection

### Evaluation of different types of mutations

The design of the three methods combining feature-based multiple models was motivated by the observation that the prediction performance of our previous work (EASE-AA) varied considerably for different types of mutations. We found that EASE-ASA (combining two models), EASE-SS (three models), and EASE-MM (consensus of the former two) can predict stability changes more accurately than both single-model methods EASE-AA and EASE-AA_2 _(Figure 3). Next, we were interested what the improvements were for different types of mutations based on the secondary structure and accessible surface area of the mutated residue. Secondary structure and solvent accessibility were calculated using DSSP [43] from the experimentally determined structures deposited in the Protein Data Bank (PDB) [44]. We also investigated prediction performance for 'small' (ΔΔ*G_u _*∈ [*−* 1, 1]) and 'large' (*|*ΔΔ*G_u_| >*1) stability changes.

**Figure 5:**
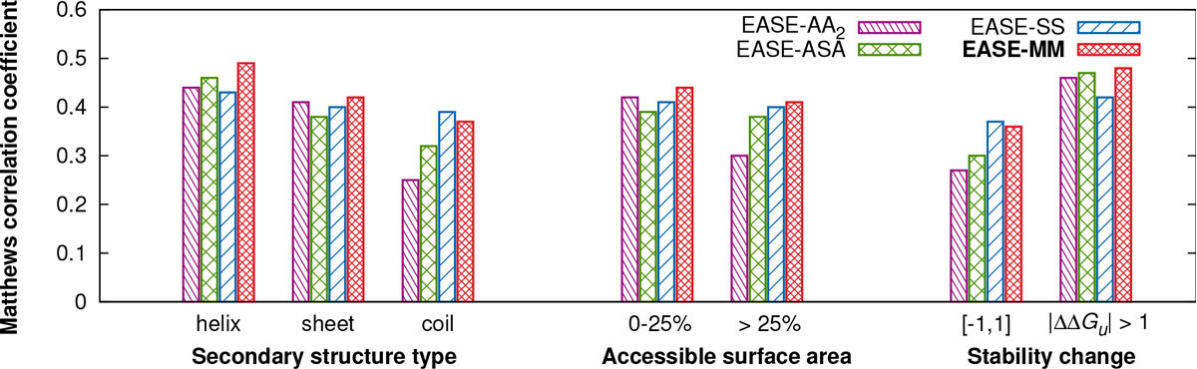
**Prediction performance of the single-model method and the three methods with multiple models for different types of mutations**. Matthews correlation coefficient (MCC) of EASE-AA_2_, EASE-ASA, EASE-SS, and EASE-MM is shown as a function of the secondary structure type of the mutated residue, relative accessible surface area of the mutated residue, and magnitude of the stability change. These are cross-validation results with the S1676 data set.

Figure 5 compares the cross-validation performance (MCC) of EASE-AA_2_, EASE-ASA, EASE-SS, and EASE-MM for different types of mutations. We analysed the performance of the methods with multiple models relative to EASE-AA_2 _because while EASE-AA_2 _encompasses only a single model, it was designed using the same feature selection procedure. Also, EASE-AA_2 _performed marginally better than EASE-AA.

Regarding the different secondary structure types, EASE-MM achieved an MCC of 0.49, 0.42, and 0.37 for the mutations in helical, sheet, and coil residues, respectively. The respective relative (absolute) improvements compared to EASE-AA_2 _were 11% (0.05), 2% (0.01), and 48% (0.12). All four methods yielded the lowest performance for the category of coil residues. However, both relative and absolute improvements of all three methods with multiple models were the highest in this category. Thus, the methods with multiple models yielded a more balanced performance for the different secondary structure types than the single-model method. This is most apparent from the performance of EASE-SS which achieved an MCC of 0.43, 0.40, and 0.39 for helical, sheet, and coil residues, respectively.

**Figure 6:**
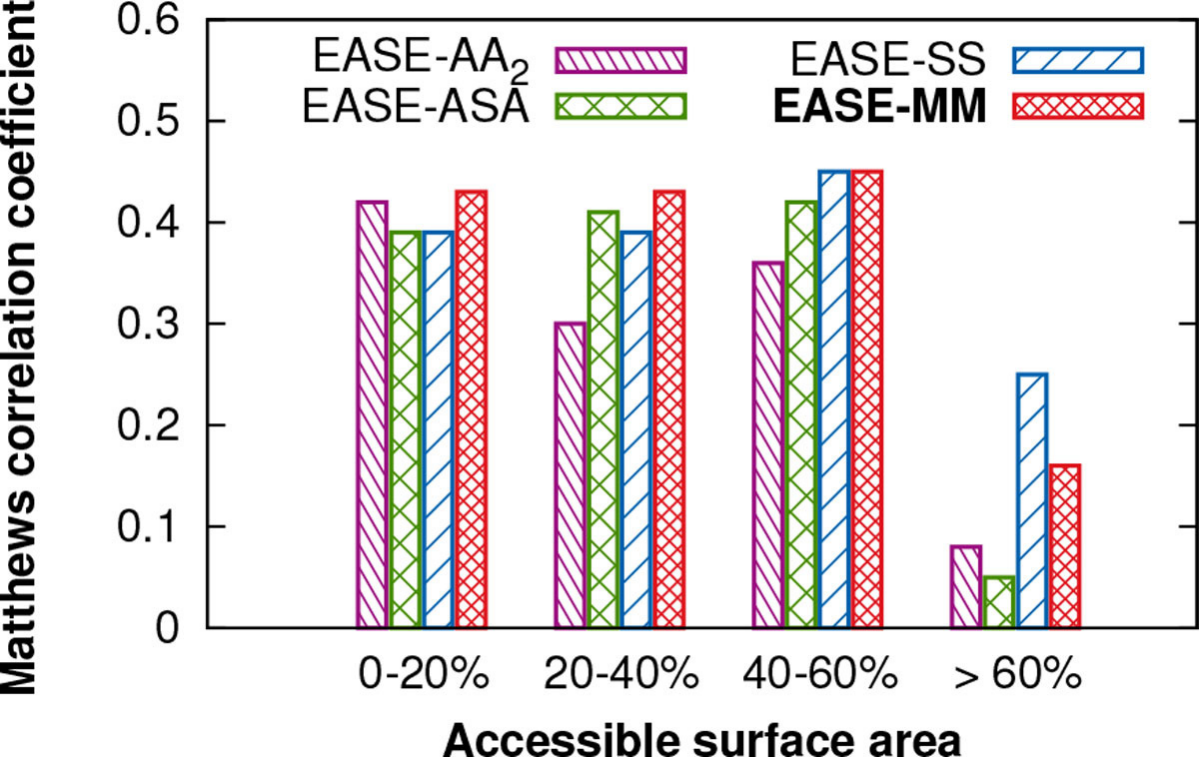
**Prediction performance of the single-model method and the three methods with multiple models for different categories of solvent accessibility**. Matthews correlation coefficient (MCC) of EASE-AA_2_, EASE-ASA, EASE-SS, and EASE-MM is shown as a function of four different categories of mutated residue's relative accessible surface area. These are cross-validation results with the S1676 data set.

Next, we analysed our results for two categories of accessible surface area (ASA) based on a threshold of 25%. We found again that EASE-MM not only outperformed EASE-AA_2 _but achieved a more balanced performance yielding an MCC of 0.44 and 0.41 for residues with ASA *≤ * 25% and *>*25%, respectively. These results constitute respective relative (absolute) improvements of 5% (0.02) and 37% (0.11) compared to EASE-AA_2_. The performance of EASE-MM in Figure 5 appears to be well balanced when we analysed only the two categories of ASA employing a threshold of 25% (the same threshold as for the design of the two models of EASE-ASA). Therefore, we were interested whether the performance varied if we considered a greater variety of ASA categories. Figure 6 shows the MCC of the compared methods as a function of four categories of ASA. The figure reveals that the performance of EASE-MM for residues more than 60% exposed to a solvent is on average 63% lower than for the other three categories covering ASA of 0-60%. While the performance of EASE-ASA was also very low in the *>* 60% exposed category (an MCC of 0.05), there was a considerable improvement in the 20-40% category. Thus, it seems that the feature selection for the EASE-ASA's *exposed *model selected mainly features which are relevant to 'partially exposed' residues. This is likely because residues with ASA *>* 60% contribute only to 7% of the S1676 data set. For the same reason (the lack of experimental data), it would not be possible to design EASE-ASA with three models including a model trained specifically for residues with ASA *>* 60%. An alternative way of improving the consensus method (EASE-MM) would be assigning a higher weight to the predicted probability by EASE-SS for mutations in residues predicted as *>* 60% exposed to a solvent. EASE-SS yielded an MCC of 0.25 in this ASA category.

Finally, we analysed performance for 'small' (ΔΔ*G_u _*∈ [*−*1, 1]) and 'large' (*|*ΔΔ*G_u_| >* 1) stability changes (Figure 5). For all four methods, the category of 'small' changes was more difficult to predict. EASE-MM reached an MCC of 0.36 and 0.48 for the 'small' and 'large' stability changes, respectively. The reason for this unbalanced performance can be twofold. Firstly, it is naturally harder to differentiate among subtle changes. Secondly, as suggested elsewhere [21,20], the strict classification of the 'small' stability changes as stabilising or destabilising can be misleading since the experimental data is affected by the error of measurement which can be as large as *±*0.48 kcal mol^*−*1^[45]. Nevertheless, the relative (as well absolute) improvement of EASE-MM (compared to EASE-AA_2_) was larger for the 'small' stability changes (33% while it was 4% for the 'large' stability changes).

Overall, EASE-ASA, EASE-SS, and EASE-MM yielded a more balanced performance for all categories of different types of mutations than the single-model method EASE-AA_2_. This result supports our hypothesis that building specialised models for different types of mutations can yield a more balanced performance. When comparing the performance of the consensus method (EASE-MM) with EASE-ASA, we found improvements in all seven categories (Figure 5). However, compared to EASE-SS, EASE-MM performed less accurately for mutations in coil residues and for 'small' stability changes. This can be attributed to relatively low performance of EASE-ASA for these two types of mutations. Because the predicted probabilities from EASE-ASA and EASE-SS contribute equally to predictions made by EASE-MM, a low performance of one of the two methods directly influences the consensus. A possible improvement would be to consider the confidence of the predicted secondary structure and use it for weighing the contribution of the probabilities predicted with EASE-SS and EASE-ASA.

### Independent test performance

**Figure 7:**
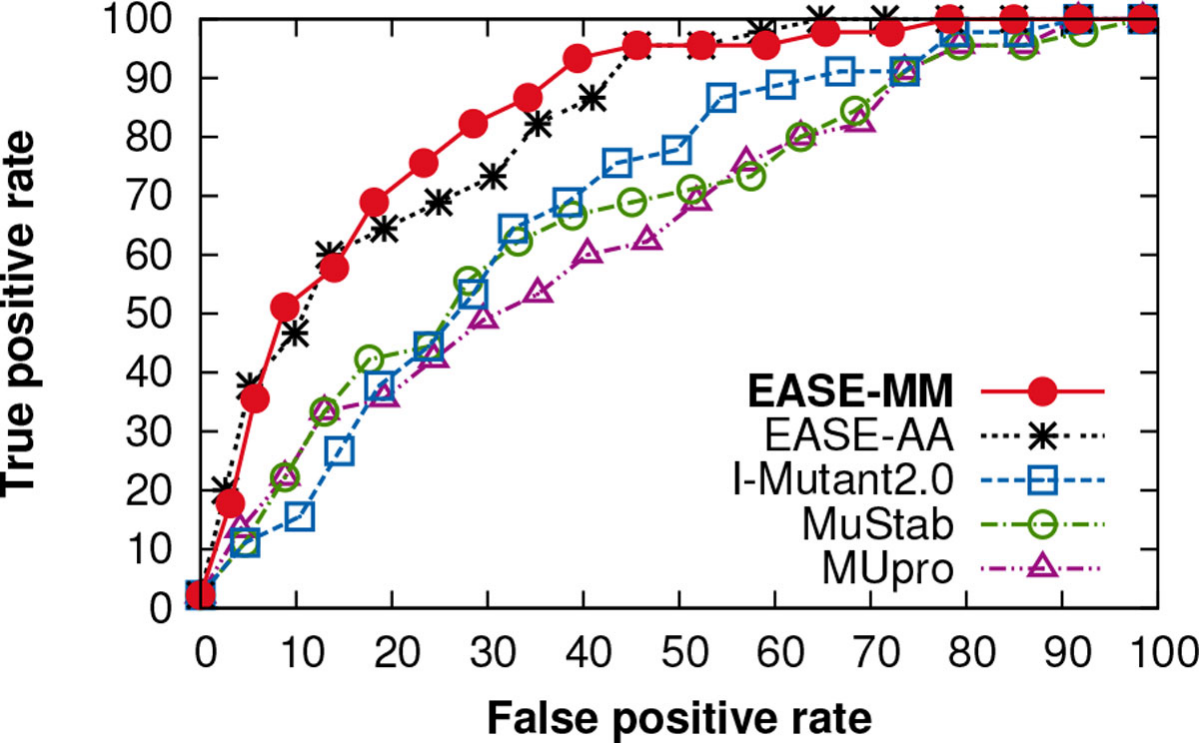
**ROC curves performance of EASE-MM, our previous work, and three currently available methods**. The true positive rate is shown as a function of the false positive rate at different prediction thresholds. These are independent test results with the S238 data set. EASE-MM, EASE-AA, I-Mutant2.0, MuStab, and MUpro achieved the area under the ROC curve (AUC) of 0.85, 0.83, 0.70, 0.67 and 0.65, respectively.

We found that EASE-MM yielded the highest cross-validation prediction performance of the five compared methods (Figure 3). However, it is important to inspect its prediction performance on an *independent *test set to see if the feature selection did not result in features which do not generalise well. Using the S238 data set, we compared the performance of three currently available methods (MUpro [18], MuStab [23], and I-Mutant2.0 [17]), our previous work (EASE-AA [26]), the single-model method (EASE-AA_2_), and the three methods with multiple models (EASE-ASA, EASE-SS, and EASE-MM). Table 3 summarises the results from the independent comparison. EASE-MM was able to considerably outperform the three currently available methods. The absolute increase in the MCC ranged from 0.20 to 0.23. The ROC curves in Figure 7 compare the true positive rate as a function of the false positive rate at different prediction thresholds. The absolute improvements in terms of the AUC for EASE-MM compared to MUpro, MuStab, and I-Mutant2.0 were 0.20, 0.18, 0.15, respectively. The performance of the three currently available methods was in agreement with the findings reported in our previous work [26]. There, we described how the evaluation is influenced when different mutations of proteins from the training set are used for testing. Since sequence similarity of the S238 data set and the data used for developing MUpro, MuStab, and I-Mutant2.0 was less than 25%, the performance of these three methods was rather low.

**Table 3:** Independent test performance (data set S238) of three currently available methods, our previous work, the single-model method, and the three methods with multiple models.

Method	AUC	MCC	Q_2_	Se	Sp	PPV	NPV
MUpro	0.65	0.24	79.41	28.89	91.19	43.33	84.62
MuStab	0.67	0.26	77.31	40.00	86.01	40.00	86.01
I-Mutant2.0	0.70	0.27	65.97	68.89	65.28	31.63	90.00
EASE-AA	0.83	0.45	82.35	60.00	87.56	52.94	90.37
EASE-AA_2_	0.72	0.36	82.77	35.56	93.78	57.14	86.19
EASE-ASA	0.81	0.43	83.19	51.11	90.67	56.10	88.83
EASE-SS	0.82	0.48	83.19	62.22	88.08	54.90	90.91
EASE-MM	0.85	0.47	81.09	68.89	83.94	50.00	92.05

EASE-MM is a consensus method of EASE-ASA and EASE-SS

The three methods with multiple models (EASE-ASA, EASE-SS, EASE-MM) yielded the AUC (MCC) of 0.81 (0.43), 0.82 (0.48), and 0.85 (0.47), respectively (Table 3). All three methods were able to considerably outperform the single-model method EASE-AA_2_. However, when compared to our previous work (EASE-AA), only EASE-MM was able to improve the AUC value (from 0.83 to 0.85). Figure 7 compares EASE-MM and EASE-AA in terms of ROC curves. For the false positive rate of 15-45%, our new method achieved a notable improvement. This is reflected in a 2% and 4% relative increase in the AUC and MCC, respectively.

We analysed why the improvements to our previous work (EASE-AA) on the S238 test set (Table 3) were not as high as for the cross-validation on the S1676 data set (Table 1). We found that there was a relative decrease of 19% in MCC on residues with accessible surface area (ASA) *≤ *25% for EASE-MM compared to EASE-AA. Coincidently, the accuracy of the predicted accessible surface area with ACCpro (decides which of the two models of EASE-ASA would be used) was 5 percentage points lower for the buried residues [a decrease from 85% (S1676) to 80% (S238)]. We conjecture that this might be one of the contributing factors to the relatively low prediction accuracy of EASE-MM on the residues with ASA *≤ * 25%. For the residues with ASA *>* 25%, EASE-MM provided a relative improvement of 50% which is in good agreement with a 46% improvement yielded in cross-validation. However, the abundance of the residues with ASA *>*25% was considerably lower in S238 (34%) compared to S1676 (48%). Therefore, despite the improvement for the *>*25% exposed residues, the overall relative increase in EASE-MM's MCC was only 4% on the S238 data set.

To confirm the significance of the improvements yielded by our new method on the S238 test set, we randomly sub-sampled the data to 119 mutations and classified the stability changes with EASE-MM and EASE-AA. We replicated this experiment 100 times. The student *t*-test's null hypothesis stated that there was no statistical difference in the MCC (AUC) between EASE-MM and EASE-AA. The *p*-values associated with this null hypothesis were less than 0.0001 for both MCC and AUC.

## Conclusions

In this work, we followed the observation that the prediction performance of our previous work [26] varies for different types of mutations based on the accessible surface area and secondary structure. We proposed a sequence-based machine learning method, EASE-MM, which predicts stability changes as a consensus of predicted probabilities of two participating methods, EASE-ASA and EASE-SS. While EASE-ASA combines two models for exposed and buried residues, EASE-SS is composed of three models for mutations in *α*-helices, *β*-sheets, and coils. Feature selection and a range of diverse features were used to design each model.

Our cross-validation results show that EASE-MM provides a notable improvement to our previous work reaching a Matthews correlation coefficient of 0.44 (Table 1). EASE-MM was able to correctly classify 73% and 75% of stabilising and destabilising protein variants, respectively, and yielded the area under the ROC curve of 0.82 (Figure 3). Using an independent test set of 238 mutations, we confirmed our results in a comparison with related work (Figure 7).

EASE-MM not only outperformed our previous work and other related methods, it achieved a more balanced results for different types of mutations based on the accessible surface area, secondary structure, and magnitude of stability changes (Figures 5 and 6). This can be attributed to using multiple models with the most relevant features selected for the given type of mutations. Therefore, our results support the presumption that different interactions govern stability changes in the exposed and buried residues [8] or in residues with a different secondary structure. Similar observations have been made about pathogenic protein variants [46]. Therefore, we aim to extend the concept of the *feature-based models *to prediction of disease-associated mutations [42,47-55] in our future work.

## Availability of supporting data

The data sets supporting the results of this article are included within the article and its additional files. The source code of our method is available for download from http://www.ict.griffith.edu.au/bioinf/ease.

## Competing interests

The authors declare that they have no competing interests.

## Author's contributions

LF designed the study, developed the methods, and conducted the data analysis under the guidance of BS. LF drafted the manuscript. BS and AS contributed to the manuscript preparation.

## Supplementary Material

Additional file 1 **Amino acid parameters**. The file containing the values of the 11 normalised amino acid parameters is available in a white-space-delimited tabular text format. Click here for file

Additional file 2 **Data sets S1676 and S238**. The files containing both the S1676 and S238 data sets are available in a white-space-delimited tabular text format. All files are compressed in a single zip archive. Click here for file

Additional file 3 **Final combinations of predictive features**. The list of the final combinations of predictive features implemented in the single model of EASE-AA_2_, two models of EASE-ASA, and three models of EASE-SS is available in PDF format. Click here for file

## Declarations

The publication costs for this article were funded by the Institute for Integrated and Intelligent Systems, Griffith University, and NICTA.

This article has been published as part of *BMC Genomics *Volume 15 Supplement 4, 2014: SNP-SIG 2013: Identification and annotation of genetic variants in the context of structure, function, and disease. The full contents of the supplement are available online at http://www.biomedcentral.com/bmcgenomics/supplements/15/S4
