# Supplementary material for: Feature-based multiple models improve classification of mutation-induced stability changes
Source: BMC Genomics. 2014 May 20;15(Suppl 4):S6. doi: 10.1186/1471-2164-15-S4-S6 (PMC4083411; doi:10.1186/1471-2164-15-S4-S6)
Supplement: Additional file 3 — Final combinations of predictive features. The list of the final combinations of predictive features implemented in the single model of EASE-AA2, two models of EASE-ASA, and three models of EASE-SS is available in PDF format. [file 1471-2164-15-S4-S6-S3.pdf]

**Table A1 Final combinations of predictive features**

| Model | Feature |
|---|---|
| EASE-$AA_2$ | SIFT score |
| EASE-$AA_2$ | $\Delta$ hydrophobicity |
| EASE-$AA_2$ | $\Delta$ isoelectric point |
| EASE-$AA_2$ | helix tendency (mean, min, max) |
| EASE-$AA_2$ | $\Delta$ compressibility |
| EASE-$AA_2$ | sheet tendency (mean, min, max) |
| EASE-$AA_2$ | $\Delta$ $M$ |
| EASE-$AA_2$ | $\Delta$ volume |
| EASE-$AA_2$ | $\Delta$ steric |
| *exposed* | $\Delta$ helix tendency |
| *exposed* | secondary structure probabilities |
| *exposed* | $\Delta$ steric parameter |
| *exposed* | SIFT score |
| *exposed* | flexibility (mean, min, max) |
| *exposed* | $\Delta$ compressibility |
| *exposed* | $\Delta$ hydrophobicity |
| *buried* | $\Delta$ hydrophobicity |
| *buried* | SIFT score |
| *buried* | $\Delta$ isoelectric point |
| *buried* | compressibility (mean, min, max) |
| *buried* | $\Delta$ $M$ |
| *buried* | helix tendency (mean, min, max) |
| *buried* | hydrophobicity (mean, min, max) |
| *buried* | polarisability (mean, min, max) |
| *buried* | relative accessible surface area |
| *helix* | $\Delta$ helix tendency |
| *helix* | $\Delta$ hydrophobicity |
| *helix* | $\Delta$ volume |
| *helix* | SIFT score |
| *helix* | volume (mean, min, max) |
| *helix* | steric parameter (mean, min, max) |
| *helix* | secondary structure probabilities |
| *helix* | disorder probability |
| *sheet* | SIFT score |
| *sheet* | $\Delta$ sheet tendency |
| *sheet* | isoelectric point (mean, min, max) |
| *sheet* | $\Delta$ volume |
| *sheet* | $\Delta$ flexibility |
| *sheet* | $\Delta$ bulkiness |
| *sheet* | ionisation (mean, min, max) |
| *sheet* | relative accessible surface area |
| *coil* | hydrophobicity (mean, min, max) |
| *coil* | $\Delta$ bulkiness |
| *coil* | relative accessible surface area |
| *coil* | SIFT score |
| *coil* | $\Delta$ polarisability |
| *coil* | $\Delta$ steric parameter |
| *coil* | $\Delta$ sheet tendency |
| *coil* | $\Delta$ hydrophobicity |

* $\Delta$ refers to the change between the introduced and deleted amino acids; (mean, min, max) was calculated for a window of six neighbouring residues
